# Copycat Layout: Network layout alignment via Cytoscape Automation

**DOI:** 10.12688/f1000research.15144.2

**Published:** 2018-08-10

**Authors:** Brett Settle, David Otasek, John H Morris, Barry Demchak

**Affiliations:** 1 Department of Medicine, University of California, San Diego, California, 92093-0688, USA; 2 University of California San Francisco, San Francisco, California, 94143, USA

**Keywords:** Workflow, Reproducibility, Cytoscape, Interoperability, REST, Microservice, Layout, Alignment

## Abstract

The copycatLayout app is a network-based visual differential analysis tool that improves upon the existing layoutSaver app and is delivered pre-installed with Cytoscape, beginning with v3.6.0. LayoutSaver cloned a network layout by mapping node locations from one network to another based on node attribute values, but failed to clone view scale and location, and provided no means of identifying which nodes were successfully mapped between networks. Copycat addresses these issues and provides additional layout options.

With the advent of Cytoscape Automation (packaged in Cytoscape v3.6.0), researchers can utilize the Copycat layout and its output in workflows written in their language of choice by using only a few simple REST calls. Copycat enables researchers to visually compare groups of homologous genes, generate network comparison images for publications, and quickly identify differences between similar networks at a glance without leaving their script. With a few extra REST calls, scripts can discover nodes present in one network but not in the other, which can feed into more complex analyses (e.g., modifying mismatched nodes based on new data, then re-running the layout to highlight additional network changes).

## Introduction

The copycatLayout app ^1^ (hereafter “Copycat”) is an evolution of the existing layoutSaver app ^2^, a visual network aligner that maps node locations from one network view (called the source) to another (called the target). Copycat aims to enable Cytoscape users to compare and contrast networks by highlighting and arranging nodes not common to both networks, and by showing both networks using the same layout, scale and placement.

We upgraded Copycat to enable Cytoscape Automation ^3^ by exposing a new REST endpoint ^4, 5^, thereby bringing the power of network-based differential analysis to automation scripts. Cytoscape Automation enables biologists to incorporate Cytoscape network visualization and analysis functionality (via REST calls) into workflows written in a language in which they are already productive (e.g., Python and R).

**Figure 1.**
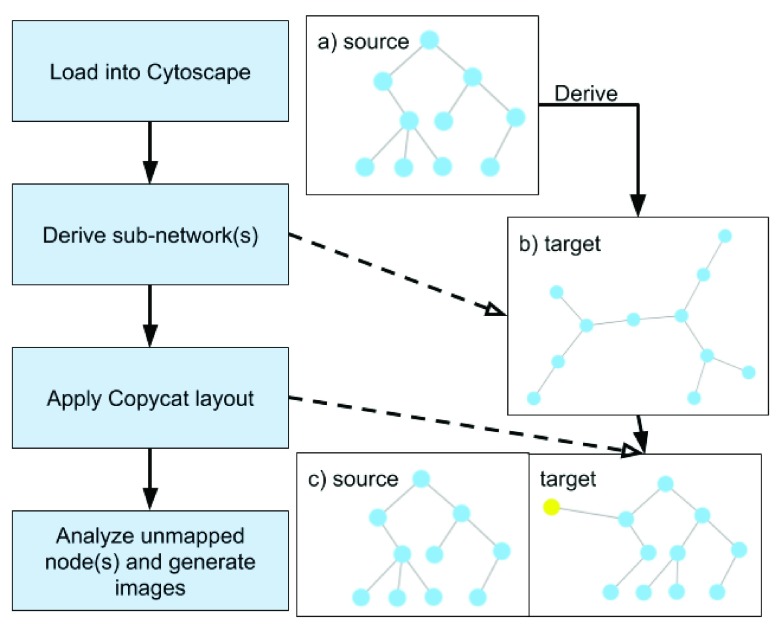
A workflow in which transformations are documented by applying Copycat. (a) The starting network is loaded into Cytoscape and a simple layout is applied. (b) Data-derived subnetwork. (c) Copycat layout is applied, mapping the starting network layout onto the subnetwork and selecting the unmapped node that was not found in the source.

For example, as Cytoscape is called to transform a network (the *target*), it’s often important to be able to show a correspondence to an original network (the *source*). The Copycat endpoint does this by invoking the Copycat layout to make common and disjoint nodes obvious and easily available to a workflow. In a workflow that performs multiple network transformations, Copycat can be used to pinpoint network differences after each transformation, thereby boosting confidence in the transformation and illuminating its real effect. Figure 1 illustrates a workflow that might call Copycat repeatedly to create a frame-by-frame movie of network manipulation.

As another example, applying Copycat layout to similar networks (i.e., rat, mouse or human) and mapping nodes by homology assignments can help verify and discover new homologous genes and gene pairs. Unmapped nodes might be useful in predicting gene positions based on successfully mapped neighbors.

In this paper, the Implementation section describes the general approach of the Copycat layout and its REST endpoint. The Operation section describes how to call the endpoint as either a Cytoscape Automation Function or Command. The Use Case section demonstrates the Copycat endpoint use in a real workflow, and the Discussion section describes the layout performance.

## Methods

### Implementation

The Copycat endpoint operates on two Cytoscape network views, where a source view contains a source network, and a target view contains a target network. The source view acts as a reference for laying out nodes in the target view.

Copycat matches nodes in a source view to nodes in a target view based on a common node attribute value. For each match, it sets the target node’s X Location, Y Location and Z Location visual attributes to those of the source node. For each source node, the attribute value is found in a column of the source network table, and likewise for each target node. (While the columns themselves may be different, they are assumed to have the same type and meaning.)

Copycat implements a multi-pass algorithm where the first pass creates a map of source node attributes to source view coordinates. The second pass searches for target node attribute values in the source node attribute map and sets the target node’s view coordinates when an attribute match is found.

A node attribute value in the source column is considered to match a value in the target column if they are lexicographically equal. Note that a poor choice of mapping attributes can lead to confusing layouts. For example, when multiple target nodes match a single source node, all of the matching target nodes will be stacked on top of each other at the coordinates of the source node. Also, if a target node matches multiple source nodes, only the last encountered source mapping is used. To avoid visual confusion, avoid having multiple nodes associated with the same attribute value. (A good choice for a mapping attribute is the node’s name if each node has a different name. A bad choice could be a node’s in-degree if multiple nodes have the same in-degree.)

**Figure 2.**
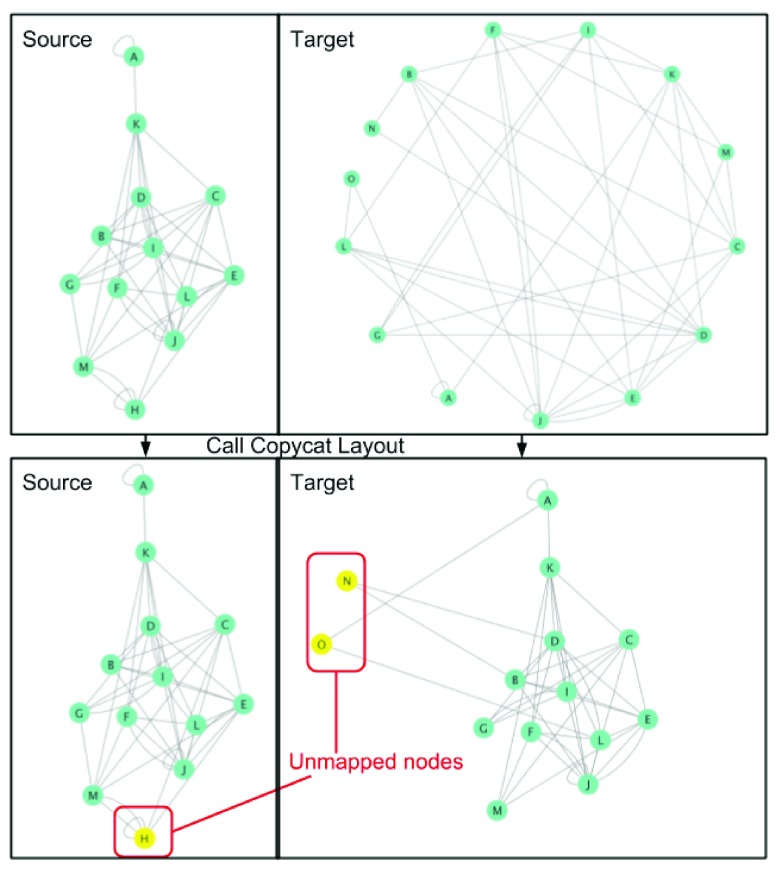
Calling Copycat layout on a derived subnetwork with the *selectUnmapped* parameter set to false. After the Copycat layout, the target network nodes have the same coordinates as the corresponding source network nodes. Because the node H in the source and nodes N and O in the target are unmatched, they are selected.

Optionally, Copycat sets the *selected* attribute for source nodes that aren’t mapped and for target nodes whose attributes aren’t found in the source node map, as shown in Figure 2. (The caller can subsequently query and act upon selected source and target nodes using other CyREST endpoints). Also optionally, unmapped target nodes are laid out in a grid in the top right portion of the target view.

### Operation

The Copycat Layout is accessible in the Cytoscape Desktop interface via the Layouts>Copycat Layout menu action, and is enabled if two or more networks exist in the network panel. For example, if the user has spent considerable time optimizing the layout of a network only to find that the data has changed, they can reload the data in the same Cytoscape session and use Copycat to copy the layout to the new network. To apply the layout, choose the source and target network views and their respective mapping columns (as defined in the *Implementation* section) and check boxes for selecting and gridding unmapped nodes if desired. The rest of this paper is intended for Cytoscape workflow programmers hoping to automate network view alignment via the Copycat layout algorithm.

Copycat exposes a single endpoint with both a Commands and Functions ^6^ variant, each with similar parameters. While both variants perform the same layout, Commands are most conveniently used in conjunction with the Cytoscape scripting facilities, and Functions are most convenient for calls from scripting languages such as Python and R.

Generally, the caller specifies the Cytoscape views containing the source and target networks (called **sourceViewSUID** and **targetViewSUID**) and provides the name of the mapping column in the source network (called **sourceColumn**) and target network (called **targetColumn**). It can control whether unmapped nodes are selected (called **selectUnmapped**) or the unmapped nodes are laid out in a grid (called **gridUnmapped**).

Note that the caller can determine columns available for a network via a CyREST endpoint, such as **/v1/networks/{networkId}/tables/defaultnode/columns**.

Note that the source and target columns must both be either string types or integer types.

The Copycat endpoint returns a CIResponse ^7^ according to Cytoscape Automation best practices. If the call succeeds, the CIResponse contains a layout result object (as the **data** element); otherwise, it contains an explanation of the error (as the **errors** element):

     {
       "data": {"mappedNodeCount": integer,
                "unmappedNodeCount": integer
       },
       "errors": []
     }

The **mappedNodeCount** value contains the number of target nodes successfully mapped, and the **unmappedNodeCount** contains the number of target nodes that did not correspond to a source node. To calculate the number of unmapped source nodes, the user can execute a GET request at **/networks/{networkSUID}/nodes/selected** and get the length of the list returned.

If either of the network views cannot be resolved by the given SUIDs or the mapping columns cannot be found in the node table of the source and target network, the **errors[0].status** element returns 404, and the remainder of the **errors[0]** element contains additional information.

To apply Copycat Layout to a network, you must be running Cytoscape version 3.6.0 or later with at least 512MB of free memory.

***Copycat as a Function.*** The Functions endpoint is documented in a Swagger page in the **Layouts** section available via **Help → Automation → CyREST API**.

The caller must pass the **SourceViewSUID** and **targetViewSUID** parameters as part of the URL ( **/v1/apply/layouts/copycat/{sourceViewSUID}/{targetViewSUID}**), and the remaining parameters in a JSON payload object:

     {
       “sourceColumn”: string, // *defaults to* “*name*”
       “targetColumn”: string, // *defaults to* “*name*”
       “selectUnmapped” : boolean,
       “gridUnmapped” : boolean
     }

Note that unlike other layout REST endpoints, the Copycat Function requires an HTTP PUT request because it consumes a JSON payload with extra parameters -- the other CyREST layout endpoints require GET requests with optional parameters directly in the URL.

Note that the **sourceViewSUID** and **targetViewSUID** must be either integers or the word “current” (meaning the view currently selected in Cytoscape). The caller can determine a network view’s SUID via a number of CyREST endpoints, including **/v1/networks/views/currentNetworkView**. The **sourceViewSUID** and **targetViewSUID** must reference different views.

Example code is provided in R, Python and as a Bash curl, but can easily be adapted into any language that supports REST calls. (Note that you must determine the values of sourceViewSUID and targetViewSUID independently ahead of these calls.)

**R**

# Basic settings for cyREST
port.number = 1234
base.url = paste("http://localhost:", toString(port.number), "/v1", sep="")

# Send it to Cytoscape!
copycat.url = paste(base.url, "apply", "layouts", "copycat",
sourceViewSUID, targetViewSUID, sep="/")
copycat.args = list(sourceColumn="name", targetColumn="name",
selectUnmapped=TRUE)
copycat.JSON = toJSON(copycat.args)
res <- PUT(url=copycat.url, body=copycat.JSON, encode="json")

**Python**

import requests, json
resp = requests.put("localhost:1234/v1/apply/layouts/copycat/{}/{}".format(sourceViewSUID, 
targetViewSUID), data=json.dumps(data))
resp = resp.json()

**Bash**

curl -X PUT --header 'Content-Type: application/json' --header 'Accept:
application/json' -d '{ \
   "sourceColumn": "name", \
   "targetColumn": "name", \
   "selectUnmapped": true, \
   "gridUnmapped": false \
 }'
'http://localhost:1234/v1/apply/layouts/copycat/${sourceViewSUID}/${targetViewSUID}'

***Copycat as a Command.*** The Commands version is documented in the Swagger page reachable from Cytoscape’s **Help → Automation → CyREST Command API** and can be found in the **layouts** section. Commands are executable via the Cytoscape Command Tool ^8^ as well as the CyREST Commands API.

The caller must pass the **sourceViewSUID** and **targetViewSUID** as part of the payload object instead of the URL. They must be the name of a network (e.g., “galFiltered.sif”) instead of an SUID integer; Copycat will operate on the primary view for the network.

Note that like other layout Command REST endpoints, the Copycat Function requires an HTTP POST request.

Note that with the current CyREST Commands, it is difficult to query Cytoscape to find the name of a network. The caller must know the name in advance, likely as a result of creating or naming it by using a different Command prior to the Copycat call (e.g., **/v1/commands/network/create empty**).

Example code is provided in R, Python and as a Bash curl, but can easily be adapted into any language that supports REST calls. (Note that you must determine the values of **sourceNetworkName** and **targetNetworkName** independently ahead of these calls.)

**R**

# Basic settings for cyREST
port.number = 1234
base.url = paste("http://localhost:", toString(port.number), "/v1", sep="")

# Send it to Cytoscape!
copycat.url = paste(base.url, "commands", "layout", "copycat", sep="/")
copycat.args = list(sourceNetwork="sourceNetworkName", sourceColumn="name", 
targetNetwork="targetNetworkName", targetColumn="name", selectUnmapped=TRUE)
copycat.JSON = toJSON(copycat.args)
res <- PUT(url=copycat.url, body=copycat.JSON, encode="json")

**Python**

import requests, json
resp = requests.put("localhost:1234/v1/commands/layout/copycat", data = json.dumps(data))
resp = resp.json()

**Bash**

curl -X POST --header 'Content-Type: application/json' --header 'Accept:application/json' -d '{\ 
   "gridUnmapped": "true", \ 
   "selectUnmapped": "true", \ 
   "sourceColumn": "name", \ 
   "sourceNetwork": "sourceNetworkName", \ 
   "targetColumn": "name", \ 
   "targetNetwork": "targetNetworkName" \ 
 }' 'http://localhost:1234/v1/commands/layout/copycat'

Additionally, the Copycat Command can be executed directly via the Cytoscape Command Tool or as part of a Cytoscape Command script as shown below, where each parameter is specified using a key-value pair. Optional parameters can be specified by appending additional key-value pairs.

layout copycat sourceNetwork={sourceNetworkName} targetNetwork={targetNetworkName}

## Use case

Many biological analyses rely on performing differential analysis on derived subnetworks. In the case of inferring gene regulatory networks ^9^, it is beneficial to show mutations at each time-step without touching the unchanged parts of the network. Identifying and visualizing the changes over time is much easier with Cytoscape Automation. After loading networks into Cytoscape, the CyREST API can be used to fetch the SUID of networks and views, and the names of columns in the node table. What follows are some of the essential steps for this kind of analysis.

# Retrieve network and view SUID from Cytoscape in Python
REST_PORT='1234' # Set to whatever rest.port is in Cytoscape preferences
REST_ENDPOINT = "http://localhost:{}/v1".format(REST_PORT)

resp = requests.get(REST_ENDPOINT + "/networks")
network_suids = resp.json()
suid_map = {'source': {'suid': network_suids[0]}, 'target': {'suid':network_suids[1]}}

resp = requests.get("{}/networks/{}/views".format(REST_ENDPOINT,
suid_map['source']['suid']))
suid_map['source']['view_suid'] = resp.json()[0] # only one view exists

Insert the network view SUIDs into the sample scripts listed above to apply the Copycat layout, and the CyREST API will return the number of mapped and unmapped nodes in the target layout. Once the layout has completed, add a GET request to **/networks/{SUID}/views/{viewSUID}.png** to generate images of the aligned networks. Adding the **selectUnmapped** parameter and a few calls to the core Cytoscape network API suite allows callers to take full advantage of the layout results by getting unmapped node information.

resp = requests.get("{}/networks/{}/nodes/selected".format(REST_ENDPOINT,networkId))
nodes = []
for nodeId in resp.json():
    resp = requests.get("{}/networks/{}/nodes/{}".format(REST_ENDPOINT,networkId, nodeId))
    nodes.append(resp.json())

The complete working example of this workflow is available in a Jupyter notebook found in the copycat-layout Github repository at https://github.com/cytoscape/copycat-layout/blob/master/notebooks/Copycat%20Automation%20Example.ipynb.

## Discussion

Using a hashmap to store node attributes and locations within solely the source network allows Copycat layout to run in O(N+M) time while only requiring O(N) memory, where N is the number of nodes in the source network and M is the number of nodes in the target network. Note that the layout performance is independent of the number of edges in either network, as edges are not accounted for in this algorithm.

It is also important to note that the Commands endpoint is automatically generated by Cytoscape Tunables and is meant to be used via Cytoscape Command Tools, whereas the CyREST Functions are crafted by the developer specifically for use in Python- and R-based automation scripts. For this reason, the Functions endpoint will be better documented, provide better errors, and accept more script-friendly parameters, such as SUIDs.

## Future plans

The Copycat API follows the same Semantic Versioning ^10^ best practices established by Cytoscape and CyREST, and future modifications will respect the same parameters, functionality, and expected outputs specified in this paper. CopycatLayout aims to be an integral tool for layout alignment in Cytoscape, and intends to accommodate future expectations of alignment analysis without overcomplicating the interface. By integrating repetitive use cases into the API (e.g., returning the list of unmapped node SUIDs as well as their count), we can provide better tools to researchers and greatly reduce the complexity of automation scripts.

We plan to implement better handling of a non-unique mapping attribute column to provide the user with more control over node pairings that are not one-to-one. For example, we could provide a dialog for viewing and managing complex mappings. Another point of interest in visual alignments is identifying overlapping edges between networks. We hope to address both of these concerns in future versions of Copycat layout.

## Summary

In this paper, we presented the Copycat layout, a network differential analysis app for Cytoscape 3. Copycat provides basic visual alignment functionality that will open the door to more data-centric alignment algorithms.

Copycat layout is also exposed via the Cytoscape CyREST API for automation users and scripted analyses. Copycat can be combined with other layouts and CyREST calls to better understand network transformations, and generate clean network difference figures.

## Data availability

The data referenced by this article are under copyright with the following copyright statement: Copyright: © 2018 Settle B et al.

Data associated with the article are available under the terms of the Creative Commons Zero "No rights reserved" data waiver (CC0 1.0 Public domain dedication). 

All data underlying the results are available as part of the article and no additional source data are required.

## Software availability

**The copycatLayout app is delivered as a component of Cytoscape starting with v3.6.0, and is also available on the Cytoscape App Store:** http://apps.cytoscape.org/apps/copycatLayout.

**Source code available from:** https://github.com/cytoscape/copycat-layout.

**Archived source code at time of publication:** https://doi.org/10.5281/zenodo.1287307 ^11^.

**License:** GNU Lesser General Public License v2.1.
