# Supplementary material for: Copycat Layout: Network layout alignment via Cytoscape Automation
Source: F1000Res. 2018 Aug 10;7:ISCB Comm J-822. Originally published 2018 Jun 21. [Version 2] doi: 10.12688/f1000research.15144.2 (PMC6039917; doi:10.12688/f1000research.15144.2)
Supplement: Peer review history

# Peer review: Referee response for version 2

**DOI:** 10.5256/f1000research.17329.r36982

**Published:** 2018-08-10

**Authors:** Bernhard Mlecnik

**Affiliations:** 1 Laboratory of Integrative Cancer Immunology, Cordelier Research Center, Paris, France; 2 Inovarion, Paris, France

All OK for me.

I have read this submission. I believe that I have an appropriate level of expertise to confirm that it is of an acceptable scientific standard.

# Peer review: Referee response for version 1

**DOI:** 10.5256/f1000research.16498.r35315

**Published:** 2018-07-09

**Authors:** Katja Luck

**Affiliations:** 1 Center for Cancer Systems Biology (CCSB), Dana–Farber Cancer Institute, Boston, MA, USA; 2 Department of Cancer Biology, Dana–Farber Cancer Institute, Boston, MA, USA; 3 Department of Genetics, Harvard Medical School, Boston, MA, USA

The article Copycat Layout: Network layout alignment via Cytoscape Automation describes an evolution of an existing Cytoscape App that allows the mapping of the layout of one network onto another network by matching the nodes in both networks using a common node attribute. As a frequent Cytoscape user, I have very much awaited for such a Cytoscape functionality to be developed as it clearly fills a gap.

 For programmers and frequent users of the Cytoscape automation functions, the article is very understandable and provides all necessary information for judging whether it suits the user's needs and how to apply it. However, for non-computational users of Cytoscape, some more information could be provided helping them to know whether it solves their needs and how to use it. I might have missed it but I cannot locate information within the article or from a reference or link where to find information how and if the App can be used within Cytoscape without using the REST API or the command line tools of Cytoscape. If it is missing, it might add value to the article to provide such an example. For example, a common user scenario that I have already encountered many times in collaborations with biologists is that we had manually optimized the layout of a network (a very time-consuming procedure) to then later realize that the network data has changed again. This meant to reload the new network and start from scratch the manual layout optimization. I understand that with the Copycat App, the layout from the previous network (in this article corresponding to the source network) can be transferred to the new network (target network) solving the aforementioned problem. It might be helpful to many readers to quickly see from within the article or elsewhere in the documentation or tutorial how to solve this problem with this new App without using scripts.

 The whole article is written in very technical terms. Even though for most of them references are provided, the article and software might be more accessible and therefore more used, especially by non-programmers, if efforts were taken to reduce some of the technicalities in the main text.

 A suggestion for future improvements of the App is the possibility to highlight differences in links between the source and target network. A nice example for such a use case is shown in Figure 1 of the paper. The networks differ by one node as highlighted by the select tool, however, the networks also differ by some links connecting nodes that are present in both networks. These link differences can be easily missed by visual inspection, especially if the networks contains more nodes than in this example, but might be extremely valuable to spot or identify by the user. I think highlighting unique links to both networks would be a great plus.

I have read this submission. I believe that I have an appropriate level of expertise to confirm that it is of an acceptable scientific standard.

# Response

**Published:** 2018-08-02

**Authors:** Brett Settle

**Affiliations:** UCSD Ideker Lab, USA

We would like to thank the referee for positive feedback and helpful comments. Below we acknowledge the notes mentioned in the review:

- This paper was published as part of a collection focused on Cytoscape Automation via CyREST, and so we did not mention applying the copycat layout via Cytoscape Desktop. A short section will be added at the beginning for non-REST users, and it will present the very use case you suggest.
- This is a very helpful note for future papers on Automation and we will take into account the technical terms that are used.
- We very much anticipate an interest in network comparison highlighting of non-matching edges as well as nodes. This will definitely be a feature in a future version of the app. Thank you for the very helpful use case.

# Peer review: Referee response for version 1

**DOI:** 10.5256/f1000research.16498.r35312

**Published:** 2018-07-04

**Authors:** Bernhard Mlecnik

**Affiliations:** 1 Laboratory of Integrative Cancer Immunology, Cordelier Research Center, Paris, France; 2 Inovarion, Paris, France

Short summary

 The Copycat Cytoscape layout plugin presented here maps the node locations of a source network to the node locations of a target network and handles missing nodes. Important is that the source node ids uniquely match the target node ids to avoid network inconsistencies after applying the layout.

 The Copycat layout is a straightforward to use plugin. The application is simple but powerful and Cytoscape needs this kind of layout algorithm.

 It is also important the the developers offer a REST enabled service to this plugin which is thoroughly described for different programming languages in the article.

 The only point the authors could address is a more sophisticated way of handling node inconsistencies. E.g. give a name/id list of nodes that are not unique in the target network to inform the user about this problem instead of just overlaying these nodes on the target network.

 Will the authors of this plugin consider edge information in the future? E.g. to give an option to map only nodes that are linked in the source and target network.

I have read this submission. I believe that I have an appropriate level of expertise to confirm that it is of an acceptable scientific standard.

# Response

**Published:** 2018-08-02

**Authors:** Brett Settle

**Affiliations:** UCSD Ideker Lab, USA

We would like to thank the referee for the positive feedback and respond to the referee’s comments:

- In the current version, a non-unique attribute column used as the mapping variable should be handled better in future versions of the app. Currently we are deciding how best to deal with these mappings.
- We would be very interested in adding functionality to restrict layout mapping to edges instead of nodes, given use cases and/or expected results.  These more difficult alignments will be considered for future releases.
